# Transcription of a protein-coding gene on B chromosomes of the Siberian roe deer (*Capreolus pygargus*)

**DOI:** 10.1186/1741-7007-11-90

**Published:** 2013-08-06

**Authors:** Vladimir A Trifonov, Polina V Dementyeva, Denis M Larkin, Patricia CM O’Brien, Polina L Perelman, Fengtang Yang, Malcolm A Ferguson-Smith, Alexander S Graphodatsky

**Affiliations:** 1 Institute of Molecular and Cellular Biology SВ RAS, Novosibirsk, Russia; 2 Institute of Biological, Environmental and Rural Sciences, Aberystwyth University, Aberystwyth, UK; 3 Cambridge Centre for Comparative Genomics, Department of Veterinary Medicine, University of Cambridge, Cambridge, UK; 4 Wellcome Trust Sanger Institute, Wellcome Trust Genome Campus, Hinxton, UK

**Keywords:** B chromosomes, Segmental duplications, Gene duplications, Karyotype evolution, Cervidae

## Abstract

**Background:**

Most eukaryotic species represent stable karyotypes with a particular diploid number. B chromosomes are additional to standard karyotypes and may vary in size, number and morphology even between cells of the same individual. For many years it was generally believed that B chromosomes found in some plant, animal and fungi species lacked active genes. Recently, molecular cytogenetic studies showed the presence of additional copies of protein-coding genes on B chromosomes. However, the transcriptional activity of these genes remained elusive. We studied karyotypes of the Siberian roe deer (*Capreolus pygargus*) that possess up to 14 B chromosomes to investigate the presence and expression of genes on supernumerary chromosomes.

**Results:**

Here, we describe a 2 Mbp region homologous to cattle chromosome 3 and containing *TNNI3K* (partial)*, FPGT, LRRIQ3* and a large gene-sparse segment on B chromosomes of the Siberian roe deer. The presence of the copy of the autosomal region was demonstrated by B-specific cDNA analysis, PCR assisted mapping, cattle bacterial artificial chromosome (BAC) clone localization and quantitative polymerase chain reaction (qPCR). By comparative analysis of B-specific and non-B chromosomal sequences we discovered some B chromosome-specific mutations in protein-coding genes, which further enabled the detection of a FPGT-TNNI3K transcript expressed from duplicated genes located on B chromosomes in roe deer fibroblasts.

**Conclusions:**

Discovery of a large autosomal segment in all B chromosomes of the Siberian roe deer further corroborates the view of an autosomal origin for these elements. Detection of a B-derived transcript in fibroblasts implies that the protein coding sequences located on Bs are not fully inactivated. The origin, evolution and effect on host of B chromosomal genes seem to be similar to autosomal segmental duplications, which reinforces the view that supernumerary chromosomal elements might play an important role in genome evolution.

## Background

B chromosomes are traditionally classified as a special class of eukaryotic chromosomes different from both autosomes and sex chromosomes [1]. A characteristic trait of B chromosomes is their dispensability; that is, there may be some individuals lacking them in the population [2]. The number of B chromosomes may vary between cells, tissues, and individuals within a population, which is due to their irregular meiotic and mitotic behavior [3]. B chromosomal meiotic drive has been detected in many plant and animal species (reviewed in [2]) and it plays a key role in the maintaining of B chromosomes through generations. The molecular mechanisms responsible for this drive remained unknown for a long period of time, but recently a non-disjunction of B chromatids accompanied by centromere activity was demonstrated in the male gametophyte of rye [4]. Although it is widely accepted that B chromosomes are highly heterogeneous and may have different properties in different organisms, until recently the concept of the totally heterochromatic and transcriptionally inert nature of B chromosomes has prevailed. Indeed, supernumerary chromosomes of various organisms often contain tandemly arranged [5] and dispersed [6] repetitive elements, interstitial telomeric sequences [7,8], ribosomal DNA clusters [9,10], histone genes [11] and so on. The first protein-coding genes were found in B chromosomes of the fungus *Nectria haematococca*[12]. Recently, a growing body of evidence has accumulated on the presence of coding genes [13-17] and even organellar genome sequences [17] on B chromosomes of different animal and plant species. This has corroborated the idea of a complex mosaic structure of supernumerary chromosomes, representing autonomous blocks of segmental duplications and heterochromatin [18]. Transcription of ribosomal genes has been detected in some plants [19] and animals [10,20]. However, no transcription of B-specific protein-coding genes has been detected in vertebrates so far.

The Siberian roe deer (*Capreolus pygargus* (CPY), 2n = 70 + 1–14 Bs) is a widely distributed cervid species inhabiting wide areas of Asia from the Volga River to the Pacific coast. The Siberian roe deer is closely related to the European roe deer (*Capreolus capreolus,* 2n = 70), but these two species do have some morphological and karyological differences and are well resolved in molecular phylogenies [21,22]. The only derived karyotypic character of the Siberian roe deer is the presence of B chromosomes [23], the number of supernumerary elements usually varies both between individuals and between cells within the same individual albeit some individuals may possess a stable number of Bs [24]. Recently, we have studied the standard karyotype of the Siberian roe deer by comparative chromosome painting and found it to be highly conserved and similar to that of the cervid ancestor [25]. Although some authors proposed the classification of several subspecies of *C. pygargus*[26,27], our studies of the mitochondrial DNA control region in the populations of extant and ancient roe deer failed to reveal any subspecies structure [28].

Here, we report on three protein-coding genes that are located within the duplicated autosomal regions on the B chromosomes of the Siberian roe deer (*Capreolus pygargus*) and demonstrate the transcription of a B-specific protein-coding sequence in fibroblast culture.

## Results

### Generation of Siberian roe deer B chromosome-specific cDNA library (CBCL)

**Figure 1:**
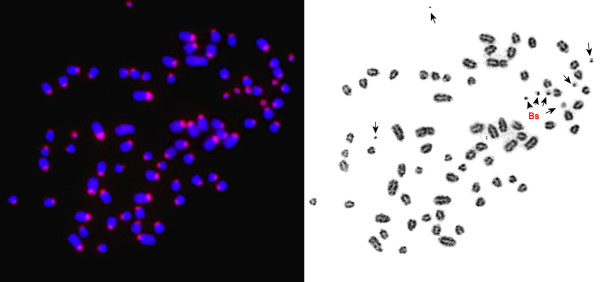
**Fluorescence *in situ *hybridization (FISH) using a B-specific flow sorting derived library on chromosomes of the Siberian roe deer.** Arrows indicate B chromosomes.

**Figure 2:**
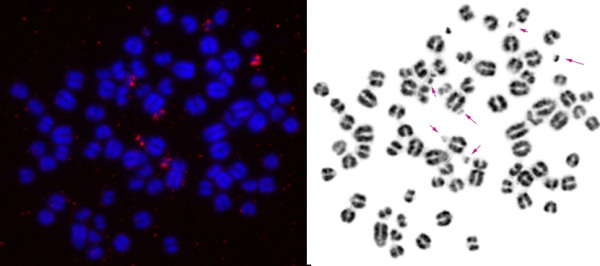
**Fluorescence *in situ *hybridization (FISH) using a B-specific cDNA library on chromosomes of the Siberian roe deer.** Arrows indicate B chromosomes.

For chromosome-specific cDNA selection we used a protocol based on the previously published selection of hybrids by affinity capture (SHAC) [29] technique with some modifications. A biotinylated library derived from degenerate oligonucleotide-primed polymerase chain reaction (DOP-PCR) amplification of flow-sorted B chromosomes was used as ‘target DNA’. We confirmed the B chromosomal origin of the biotinylated library by Fluorescence *in situ* hybridization (FISH) without Cot5 suppression. Signals were detected on all eight B chromosomes of the Siberian roe deer (CPY_d specimen, Table 1) (Figure 1). In addition, the subcentromeric heterochromatic blocks on all autosomes (but not on X chromosomes) were painted, suggesting the presence of shared heterochromatic sequences on all autosomes and B chromosomes. No signals on autosomal arms were detected. The ‘source’ cDNA was synthesized from total RNA isolated from the CPY_d fibroblast culture (passage 6); the cDNAs of the ‘source’ library were flanked by primers (provided in the library construction kit). We used an excess of CPY Cot5 DNA in hybridization experiments to reduce the presence of the repetitive DNA in the final B-specific cDNA library. The quality of the CBCL (in terms of chromosome specificity and depletion of the repetitive DNA) was confirmed by FISH (Figure 2). Noteworthy were the absence of signals on autosomal pericentric heterochromatic blocks and a high specificity of the resulting B-specific probe.

**Table 1:** Characteristics of animal samples used in the study

**Abbreviation**	**Species**	**Locality**	**No. of B chromosomes**	**Sex**
AAL	*Alces alces*	Yakutia region, Russia	0	Male
BTA	*Bos taurus*	Novosibirsk region, Russia	0	Female
CEL	*Cervus elaphus sibiricus*	Altai region, Russia	0	Unknown
CPY_a	*Capreolus pygargus*	Novosibirsk region, Russia	0	Male
CPY_b	*C. pygargus*	Altai region, Russia	4	Male
CPY_c	*C. pygargus*	Altai region, Russia	8	Female
CPY_d	*C. pygargus*	Altai region, Russia	8	Female
CCA_a	*Capreolus capreolus*	UK	0	Unknown
CCA_b	*C. capreolus*	Leningrad Oblast, Russia	0	Unknown
MGO	*Mazama gouazoubira*	Berlin Zoo, Germany	3	Male

### B-specific cDNA cloning and sequencing

**Figure 3:**
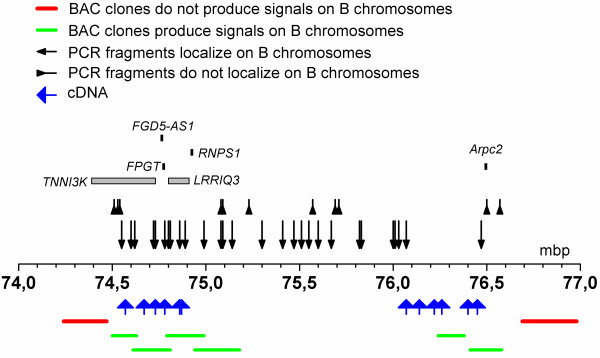
**Fragments mapped on B chromosomes of the Siberian roe deer by cDNA sequencing, cattle bacterial artificial chromosome (BAC) localization and polymerase chain reaction (PCR)-assisted mapping.** BTA3 is the segment of cattle chromosome 3 from the Btau_4.6.1.

To characterize the CBCL and estimate its content, we cloned the PCR products in plasmid vectors and sequenced 33 random clones. The sequences were aligned against cattle genome assemblies (UCSC genome browser on Cow, October 2011 (Baylor Btau_4.6.1/bosTau7)) using the BLAT search genome alignment tool and the following results were obtained (here we present only matches that had over 80% similarity of 150 bp): eight clones showed similarity to dispersed repetitive elements (mostly long interspersed nuclear elements (LINEs) from the L1 BT family); three clones were homologous to genes from the mitochondrial genome. Of the 22 clones that showed a high similarity to unique parts of the bovine genome (*Bos taurus* (BTA)), 18 clones were mapped to a 1.8-Mbp region of BTA3 (74.6 to 76.4 Mbp according to (UCSC genome browser on Cow, October 2011 (Baylor Btau_4.6.1/bosTau7))) (Table 2, Additional file 1) (GenBank: JN871269 to JN871285). The remaining four unique clones were homologous to regions on BTA 2, 7, 10 and 28 (data not shown). The cDNAs homologous to the BTA3 region were not randomly distributed across the 1.8 Mbp genomic segment; 2 areas of high match density were observed: 74.6 to 74.9 Mbp (7 matches) and 76.0 to 76.4 Mbp (11 matches), separated by a 1 Mbp region lacking any matches (Figure 3).

**Table 2:** The characteristics of sequenced B chromosome-specific cDNA clones

**Name of cDNA clone**	***Bos taurus *(BTA) chromosome**	**Homology to BTA segment, %**	**Length, bp**	**Gene**	**Location of homologous region on BTA3, bp**	**GenBank accession no.**
c7g	3	90.3	440	*TNNI3K* (intron)	74,572,819 to 74,573,252	JN871276
c3t	3	94.3	310	*TNNI3K* (intron)	74,666,209 to 74,666,514	JN871280
cz2	3	91.7	371	*TNNI3K* (intron)	74,727,542 to 74,727,927	JN871284
c7i	3	95.8	308	*TNNI3K* (exon) *FPGT* (exons)	74,729,423 to 74,782,291	JN871272
cz1	3	92.8	597	*LRRIQ3* (exon)	74,855,578 to 74,856,167	JN871283
c5b	3	90	331	*LRRIQ3* (intron)	74,856,714 to 74,857,042	JN871285
c9t	3	93.9	384	*LRRIQ3* (intron)	74,866,986 to 74,867,372	JN871281
c4f	3	92	595		76,073,726 to 76,074,320	JN871270
c3	3	90.8	481		76,143,925 to 76,144,402	JN871269
c6h	3	88.8	162		76,215,946 to 76,216,107	See Additional file 1
c7f	3	91.1	633		76,256,747 to 76,257,369	JN871275
c9	3	88.5	270		76,257,204 to 76,257,458	JN871273
c4b	3	88.6	586		76,260,507 to 76,261,080	JN871274
ct2	3	88.5	347		76,261,257 to 76,261,587	JN871279
c8g	3	93	322		76,261,288 to 76,261,613	JN871277
c9v	3	95.3	568		76,402,615 to 76,403,200	JN871282
c5	3	93.4	352		76,402,676 to 76,403,051	JN871271
cs9	3	91.4	405		76,448,848 to 76,449,833	JN871278

### The mapping of B chromosomes with bovine bacterial artificial chromosome (BAC) clones

To confirm the presence of a BTA3-homologous region on B chromosomes of *C. pygargus* we performed FISH of bovine BAC clones from the respective genomic segments. Based on the distribution of B chromosome-specific cDNA clones we selected sequenced bovine BAC clones from the CHORI-240 library for FISH mapping (Additional file 2: Table S1). FISH was performed on the metaphase chromosomes of *C. pygargus* with four Bs (CPY_b) and with eight Bs (CPY_d). As a control we also mapped the same BAC clones via FISH on bovine metaphase chromosomes.

**Figure 4:**
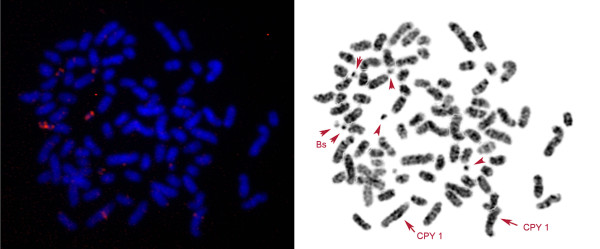
**Localization of bovine bacterial artificial chromosome (BAC) CH240-10H15 on the chromosomes of the Siberian roe deer (*Capreolus pygargus *(CPY)) with eight B chromosomes (CPY_d).** Arrows indicate signals on CPY1 and B chromosomes.

All BACs produced specific signals on the Siberian roe deer chromosome 1 and bovine chromosome 3. These results are consistent with our data of cross-species chromosome painting that demonstrated complete homology of chromosomes BTA3 and CPY1 [25]. Six bovine BAC clones (CH240-10H15, CH240-444I8, CH240-493Р4, CH240-515С3, CH240-454D22 and CH240-351I13) produced strong signals on B chromosomes of *C. pygargus*, confirming the presence of the CPY1 fragment (Figure 4). BAC clones CH240-131I21 and CH240-385G2 did not give any specific signal on B chromosomes, presumably due to the absence of the respective genomic loci on these elements, although this does not exclude the presence of a short flanking fragment from these BACs.

Some BAC clones produced signals of varying intensity on different B chromosomes suggesting molecular heterogeneity among supernumerary elements, for example, the BAC clone CH240-444I8 gave more intensive signals on six out of eight B chromosomes in the CPY_d, whereas all four B chromosomes were painted in equal intensity in the CPY_b. CH240-454D22 gave signals of different intensities on B chromosomes of CPY_b: two out of four B chromosomes consistently showed signals of two to three times stronger intensity than the remaining ones. This could reflect a different degree of regional amplification.

The localization of BAC clones further confirmed the results of cDNA mapping. Assuming that the cDNAs and BACs demonstrate the presence of a continuous segment homologous to BTA3, full copies and parts of some genes must be present in the proximal part of this segment (*TNNI3K, LRRIQ3, FPGT*, non-protein coding *FGD5-AS1*, and a pseudogene *RNPS1*), while the distal part of the segment seems to be gene sparse (it contains only a pseudogene of *Arpc2*) and represents a large gene desert (Figure 3).

### The mapping of B chromosomes by PCR

To refine the boundaries of the amplified regions on B chromosomes we used PCR-assisted mapping on the flow-sorted B chromosome library. We designed 40 primer pairs from BTA3 based on conserved regions of the bovine genome (UCSC genome browser on Cow, October 2011 (Baylor Btau_4.6.1/bosTau7)), taking into account the distribution of B chromosome-specific cDNAs and BAC mapping results (Figure 3). The PCR was carried out using the DOP-PCR library, derived from flow-sorted B chromosomes of the Siberian roe deer, as template. Parallel amplification was conducted with bovine and total roe deer genomic DNAs. The PCR products of appropriate size resulting from the B chromosome-specific library, and genomic DNA of both cattle and Siberian roe deer indicated the presence of these fragments on supernumerary chromosomes of *C. pygargus*. The absence of several closely located products in the B chromosome-specific library indicated deletions or segment boundaries. We confirmed the specificity of several PCR products by direct sequencing. This method allowed us to define precisely the boundaries of the B chromosome-specific segmental duplication. The PCR-assisted mapping extended the size of the segment on supernumerary chromosomes to approximately 2 Mbp in total, with some possible deletions in the central part of the region (alternatively, these might be due to incomplete coverage of B-specific libraries). To determine the upstream boundary of the segment we used five primer pairs. This boundary was found to be located between positions 74,538 kbp and 74,600 kbp. Using three primer pairs we mapped the downstream boundary of the segment to the interval between 76,468 kbp and 76,502 kbp.

Although the homology of the roe deer B chromosomes to each other has been demonstrated by reverse painting of probes derived from single-copy microdissection experiments (data not shown) and was implicated by the data on BAC localization, we did not have any evidence on the general structural homogeneity of B chromosomes.

### The estimation of copy number variations of the regions localized on the B chromosomes of the Siberian roe deer

To estimate the copy numbers of amplified B genes we applied qualitative PCR (qPCR) using conserved parts including the sequence of the fourth exon from *FPGT* and *LRRIQ3* and a promoter region of *TNNI3K* (Additional file 3: Figure S1). As a single copy reference we used a conserved segment of *PTGFR* gene located on bovine chromosome 3 outside the region amplified on B chromosomes of the Siberian roe deer (approximately 4.6 Mbp downstream). To achieve equal efficiency of amplification in both cattle and Siberian roe deer we designed primers based on identical short sequences in both species. The short sequences in the Siberian roe deer were obtained by targeted sequencing.

**Figure 5:**
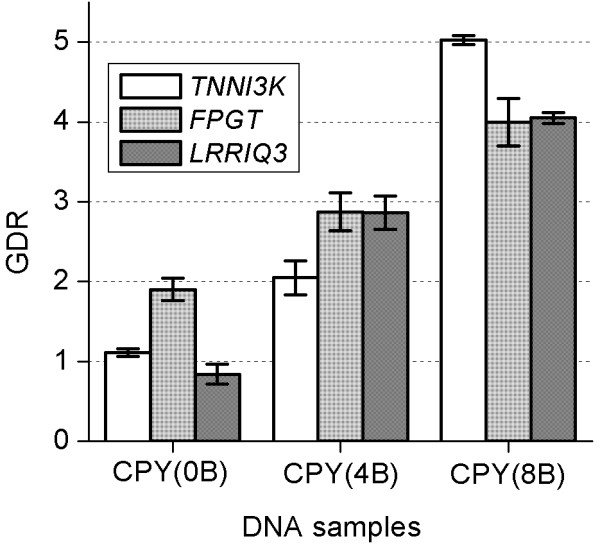
**Gene dose ratio (GDR) of *FPGT*, *LRRIQ3 *and *TNNI3K *genes in Siberian roe deer with zero, four and eight B chromosomes.** The GDR values are relative to the number of respective gene copies per haploid genome in each sample (cattle DNA was used as reference). Error bars represent the standard deviation.

We characterized the copy number of regions present on B chromosomes (Bs) in Siberian roe deer without B chromosomes (CPY_a), Siberian roe deer with four Bs (CPY_b) and Siberian roe deer with eight Bs (CPY_d) (Figure 5). The qPCR results showed that the regions of *TNNI3K* and *LRRIQ3* genes are present in two copies per diploid genome in CPY_a (similar to *PTGFR*) whereas the region of *FPGT* gene is present in four copies per diploid genome in the same animal. This indicates a duplication of the region containing *FPGT* gene in CPY_a, although no extra chromosomes were found in the specimen.

We found that the regions of *FPGT* and *LRRIQ3* genes are present in six copies per diploid genome in CPY_b (four Bs), suggesting a direct correlation between the number of extra copies of the gene fragment and the number of B chromosomes. We showed that the region of *TNNI3K* gene is present in four copies per diploid genome in CPY_b, suggesting that the copies of *TNNI3K* gene segment are localized only on two out of four B chromosomes. We found the region of the *TNNI3K* gene is present in ten copies per diploid genome in the specimen CPY_d (eight Bs) (suggesting each of B chromosomes carries a single copy of this segment). The regions of *FPGT* and *LRRIQ3* genes were found to be represented by eight copies per diploid genome in CPY_d (suggesting that at least two Bs lack the segment). Generally these findings correlate well with the results of cattle BAC clone localization and demonstrate that not all B chromosomes contain full copies of the proposed amplified fragment. Although the number of segment copies depends on the number of B chromosomes, this correlation is not necessarily direct, as some B chromosomes may lack the full fragment. Our results demonstrate some variation in the composition of B chromosomes despite overall similarity and general homology.

### DNA sequence comparison of non-B chromosomal and B chromosome-specific fragments

We used targeted sequencing to compare some homologous regions from B chromosomes and non-B chromosomes. We sequenced several regions (from 84 bp to 1,443 bp), comprising the promoter region of the *TNNI3K* gene (GenBank: JN871289 to JN871291), the first three exons of *FPGT* (Additional file 1) and the second exon of *LRRIQ3* gene (GenBank: JN871294, JN871295) in the library of flow-sorted B chromosomes as well as in the total genomic DNA of the European roe deer and several specimens of the Siberian roe deer (CPY_a-CPY_d) (GenBank: JN871289 to JN871291, JN871294, JN871295). In addition, we sequenced the fourth exon of *FPGT* gene (GenBank: JN871287, JN871288) and the first exon of *TNNI3K* gene (Additional file 1) in the European roe deer and CPY_d. We found nucleotide substitutions specific to B chromosomes in two cases. The B chromosomal second exon of *LRRIQ3* gene carries a transition (G > A) (valine141 to isoleucine141); the comparison was based on both the roe deer without B chromosomes and cattle, in the DOP-library of B chromosomes (GenBank: JN871295), in B chromosome-specific cDNA and in a fragment from total cDNA derived from CPY_d (obtained sequences were identical to GenBank: JN871295).

The B chromosomal second exon of *FPGT* carries a transition A > G (lysine83 to arginine83) (Additional file 1). The same B-specific mutation was found in the second exon of *FPGT* in a cDNA clone (described below).

It is intriguing that the first triplet of *FPGT* encodes for valine both in B chromosomes and in the genomic DNA of the Siberian roe deer with four and eight B chromosomes (CPY_b, CPY_d) (due to the use of total genomic DNA as template we could not reveal the underrepresented adenosines). A heterozygous condition (valine/methionine) was revealed in the genomes of two individuals of the European roe deer, while we found only methionine in the *FPGT* first position in the Siberian roe deer without B chromosomes (specimen CPY_a) (Additional file 1). Additional experiments are needed to test if this A1 > G1 transition might be characteristic for a pseudogene in the European roe deer, while the valine triplet (GTG) is associated with the segmental duplication of *FPGT* located on B chromosomes, since the GTG is known to be a less effective starting codon and may result in the loss of expression or N-terminally truncated proteins [30,31]. However, it is possible that an alternative upstream exon with a conserved ATG codon may be used. Interestingly the same A/G single nucleotide variation at the first position has been described in human *FPGT*[32].

### Evidence of FPGT transcription from B chromosomes

The finding of a B chromosome-specific mutation (A > G in the second exon of *FPGT*) in the processed *FPGT-TNNI3K* cDNA (GenBank: JN871272) prompted us to study this case in detail. First, we sequenced the second exon of *FPGT* in CPY_a (without Bs), in red deer (*Cervus elaphus*), gray brocket deer (*Mazama gouazoubira*), and Eurasian elk (*Alces alces*), and found the ‘wild type A’ condition to be conserved (sequencing of B-specific DOP-PCR library, genomic DNA of CPY_d-specific and B-specific cDNA revealed the derived ‘G’ condition). Comparison with many other sequenced vertebrates including opossum, platypus, rooster and anole lizard further corroborated the high conservation of the position in various phylogenetic lineages. To exclude the possibility that the mutation occurred on the autosomal copies of CPY with B chromosomes, we conducted comparative sequencing of autosomal and B chromosomal segments from the same animal. To achieve this, we microdissected separately chromosomes CPY1 and Bs (from the specimens CPY_d and CPY_c) in separate tubes and sequenced the region of interest. Four sequences derived from non-B chromosomes gave ‘a wild-type A’ condition, while three B chromosomal sequences gave ‘a derived G’ condition. To exclude a possible polymorphism among B chromosomes we sequenced ten FPGT exon 2 carrying clones derived from two different flow sorting fractions. All these clones carried the ‘G’ mutation. Based on our discovery of a processed transcript carrying the derived state (in CPY_d) we concluded that this region is transcribed not from autosomes but from B chromosomes.

## Discussion

Siberian roe deer belong to the cetartiodactyl family Cervidae, where B chromosomes have also been described in five *Mazama* species [33]. It is interesting that both *Capreolus* and *Mazama* genera likely possess identical standard karyotypes (2n = 70 + Bs) and have GC-rich small B chromosomes, which may suggest a common ancestry of supernumerary elements. However, our studies on the brown brocket deer (*Mazama gouazoubira*) did not reveal any homologous unique segments between B chromosomes of these species (unpublished data), suggesting a fairly recent and independent origin of Bs in *Capreolus* and *Mazama*. However, there could be a particular predisposition in the genome organization that underlies the higher frequency of B chromosomes appearance in Capreolinae.

Concerning the general polymorphism of B chromosomes, we should emphasize that a Siberian roe deer specimen carrying no B chromosomes (CPY_a) was described here for the first time. The finding of this animal was quite unexpected, considering the many previous karyotyping studies of Siberian roe deer, describing from 1 to 14 copies of B chromosomes per diploid genome [23,24,34,35]. The presence of an animal lacking Bs raised the question of whether this could be a misidentified *C. capreolus* individual. The analysis of the mitochondrial DNA control region allowed us to unambiguously describe the species as *C. pygargus* (the haplotype Ns99 is identical to the ancient haplotype DC6 ([28], GenBank: GU811828). This finding indicates that the amount of B chromosomes in the Siberian roe deer is highly variable between individuals (0 to 14) and that the presence of B chromosomes is not essential for animal physiology and cannot be used as a species discriminating character. It is of interest that some animals were reported as lacking the intraindividual mosaicism of Bs [24]. In this study, we have chosen animals with stable karyotypes from Altai.

The same method of cDNA-selection-based mapping of B chromosomes described here has also been applied to the red fox (*Vulpes vulpes*). In this species we mapped cDNAs to seven different autosomal regions, and these results were further confirmed by subsequent canine BAC mapping [36]. It seems that the roe deer B chromosomes contain a large segment from a single genomic locus, while canid B chromosomes have a complex structure comprising segments from multiple regions. B chromosomes of the red fox and the raccoon dog share some segments (homologous to canine chromosome 13) while most other segmental duplications (five in the red fox and three in the Chinese raccoon dog) are species specific.

It is worth mentioning that the fragment identified on Siberian roe deer B chromosomes is located near the evolutionary breakpoint on cattle chromosome 3 and contains a large gene desert conserved in both human and cattle. Moreover, we found several large segmental duplications in the cattle genomic region. This might have led to a general genomic instability of the locus and its involvement in B chromosome formation.

The finding of coding genes on B chromosomes of the Siberian roe deer further emphasizes the importance of studying regions previously believed to be non-coding. Representing a variation in number of coding and non-coding sequences these elements may influence many aspects of genome activity. Finding a significant amount of segmental duplications in many vertebrate species suggests that B chromosomes act as autonomously segregating karyotypic elements, carrying some genomic regions that could potentially influence overall genome plasticity and contribute to novel adaptations.

The genes that were identified on the Siberian roe deer B chromosomes, *TNNI3K* (partial), *FPGT* and *LRRIQ3*, may be expressing and influencing metabolism of the cells. Partial sequencing of these genes on B chromosomes and in the Siberian roe deer without any Bs has demonstrated a high sequence conservation and very low number of B-specific substitutions, which might reflect a relatively recent origin of B chromosomes in the Siberian roe deer from a large segment derived from CPY1. The B-specific transcript (GenBank: JN871272) described here represents an alternatively spliced variant, consisting of two exons of *FPGT* (the first and the second) and a large part of the second exon of *TNNI3K*. Multiple transcripts composed of different exons due to alternative splicing of *FPGT-TNNI3K* readthrough transcript have been described in human [37]. However, the combination of exons in the roe deer transcript is different from previously published. *TNNI3K* seems to be very important in cardiomyogenesis, since its expression promotes the differentiation of cardiomyocytes and enhances cardiac performance [38,39], while *FPGT* (the full copy of the gene is present on roe deer Bs) participates in reutilization of l-fucose arising from cellular metabolism of glycoproteins and glycolipids [40]. The locus including both *TNNI3K* and *FPGT* genes was found to be responsible for susceptibility to viral myocarditis in mice [41]. Transcription of truncated copies might generally affect the expression of autosomal copies, but more detailed research is needed here. However, these genes maybe partly or completely epigenetically inactivated. Thus, the finding of the B-derived transcript corroborates the hypothesis of transcriptional activity of segmental duplications. Further studies will determine if transcription of B chromosomal copies of the gene *LRRIQ3* also takes place. At least we found a putative B chromosome-specific mutation that might be used as a marker of B-derived transcripts, but have not yet excluded a possible case of autosomal heterozygosity of this mutation. It should be emphasized that the transcription of the B chromosomal gene was demonstrated only in fibroblasts and additional experiments are required to test the expression of this gene in other cells and tissues of the organism. It is also possible that cultured fibroblasts might manifest different properties and have different activity of some genes.

## Conclusions

Application of novel molecular technologies has allowed the localization of unique sequences, comprising protein-coding genes, on B chromosomes of several vertebrate species [13,14,16,36]. Use of high-throughput sequencing methods to obtain a large amount of information about the structure of whole genomes looks very promising for studying species with supernumerary chromosomes. However, our study shows that it should be supplemented by individual chromosome copy isolation and analysis to differentiate the non-B chromosomal and B-specific components. New data on supernumerary elements of the genome clearly indicate that the role of B chromosomes in evolution and their influence on the host are largely underestimated.

Considering that mammalian B chromosomes carrying additional copies of expressed genes do not have any pronounced adverse effect (at least none described so far), their use for the future development of artificial mammalian chromosomes seems promising: our studies demonstrate that they can be isolated easily from the rest of the genome by chromosome sorting, and studies of other authors show that flow-sorted chromosomes can be inserted into oocytes [42]. Employing newly developed methods of zinc finger nuclease technology [43] it would be of interest to use animals carrying B chromosomes as models for transgenic studies.

## Methods

### Ethics statement and sample collection

All Siberian roe deer, European roe deer (Leningrad oblast), Eurasian elk and red deer tissue samples were provided by local hunters; animals were not killed for the purpose of this study. Fibroblast tissue cultures of European roe deer (UK), cattle and brown brocket were taken for DNA isolation from the collection of the Comparative genomics group (Cambridge). Table 1 lists the number of specimens and their characteristics.

### Cell culture and chromosome preparation

Fibroblast cell lines were established from the ear cartilage tissue of Siberian roe deer individuals with different numbers of B chromosomes as described previously [44]. The cell lines were cultured at 37°C in Dulbecco’s modified Eagle medium (DMEM) and αMEM (Gibco, Invitrogen, Paisley, UK) enriched with 10% fetal bovine serum (Gibco), penicillin (100 units/ml) and streptomycin (100 μg/ml) or kanamycin (100 μg/ml). Before harvest, the cells were treated with 1 μg/ml of ethidium bromide (final concentration) for 2 h and treated with 0.05 mg/ml colchicine (final concentration) for 45 minutes. Preparation of metaphase chromosomes was made according to standard procedures that included a 45-minute hypotonic treatment in 0.56% KCl, fixation in 3:1 methanol/glacial acetic acid, followed by slide preparation and air drying. The number of B chromosomes was analyzed in all *C. pygargus* samples (at least 50 cells per sample) and we detected no variation in the number of B chromosomes between cells.

### Flow sorting

B chromosomes of the Siberian Roe deer (CPY_d) have been flow sorted on a FACStar Plus, Becton Dickinson, USA, as described previously [45,46]. The peak containing B chromosomes was very well resolved and distinct from non-B chromosomal peaks (Additional file 4: Figure S2). We collected B chromosomes into 4 tubes containing 20 μl of water (1,000 chromosomes per tube).

### Generation of B chromosome-specific libraries

Chromosome-specific libraries were made by DOP-PCR amplification of flow-sorted chromosomes [47]. DOP-PCR-amplified chromosome-specific DNA was labeled during the secondary PCR by incorporating biotin-16-dUTP (Roche, Switzerland) or Cy3-dUTP (GE Healthcare, UK) [48]. Chromosome specificity of the library was confirmed by FISH (Figure 1).

### Selection of B chromosome-specific cDNAs

Total RNA was isolated from several millions of fibroblasts from the sixth passage of the Siberian roe deer primary culture (CPY_d) using the MasterPure RNA purification Kit (Epicentre, UK) according to the manufacturer’s protocol. A double stranded total cDNA library was constructed using the BD SMART™ PCR cDNA Synthesis Kit (BD Biosciences) according to the manufacturer’s protocol. Two rounds of B chromosome-specific cDNA selection were conducted using a method based on previously published selection of hybrids by affinity capture (SHAC) [29]. Briefly, 50 μl of cDNA PCR library (driver) and 1 μl of biotin-labeled B chromosome-specific library mixed with 5 μg of *C. pygargus* Cot5 DNA (tracer) were denatured for 3 minutes at 95°C. After denaturing the products were adjusted with sodium dodecyl sulfate (SDS) (up to 0.1%) and saline-sodium citrate (SSC) (up to 1 ×) and prehybridized separately at 65°C for 30 minutes to suppress the repetitive elements. Both solutions were mixed together and hybridized overnight at 65°C. The resulting DNA duplexes were captured by streptavidin-coated magnetic beads, Dynabeads® M-280 Streptavidin (Invitrogen, UK) for 20 minutes at 65°C. Magnetic beads were washed twice in 2 × SSC, 0.1% SDS for 5 minutes at 65°C, five times in 0.2 × SSC, 0.1% SDS for 5 minutes at 65°C and once in 0.2 × SSC at 30°C. The cDNAs were released by denaturing the beads in Tris-ethylenediaminetetra-acetic acid (TE) buffer for 5 minutes at 95°C. Selected cDNAs were amplified by PCR with specific primer (provided by BD Bioscience). cDNAs, after the first round of selection were subjected to the second round, to increase the chromosome specificity. The quality of the final CBCLs was controlled by FISH (Figure 2).

### Cloning of cDNA fragments

The B-specific cDNA library was cloned using TOPO® TA Cloning® Kit (Invitrogen, UK) according to the manufacturer’s instructions. In all, 50 single colonies were cultured for plasmid DNA isolation; 33 clones with BD primer flanked inserts were selected for sequencing.

### Fluorescence *in situ* hybridization

Probes for fluorescence *in situ* hybridization were labeled either by direct incorporation of labeled dUTP in PCR (with DOP or BD primers) or using a nick translation kit (Invitrogen, UK) following the manufacturer’s instructions (for bovine BACs). Fluorescence *in situ* hybridization was performed using a standard protocol [49,50].

### Microscopy

Images were captured and processed using the CytoVision Genus system (Applied Imaging, USA) and a Cohu CCD camera mounted on an Olympus BX-60 microscope, or using Videotest 2.0 Image Analysis System (Saint Petersburg, Russia) and a Baumer Optronics CCD Camera mounted on Axioscope 2 plus microscope (Carl Zeiss, Germany).

### Isolation of DNA from bovine BAC clones

DNA was isolated from bovine BAC clones (Additional file 2: Table S1) of the genomic BAC library CHORI-240 using the QIAGEN Large-Construct Kit (QIAGEN, UK).

### Isolation of DNA from animal tissues and cell cultures

DNA was isolated from all samples listed in Table 1 using DNeasy Blood & Tissue Kit (QIAGEN) according to the manufacturer’s instructions.

### PCR-assisted mapping of the flow-sorted CPY B chromosome-specific library

PCR-assisted mapping was conducted using primers designed from cattle chromosome 3 region (UCSC genome browser on Cow, October 2011 (Baylor Btau_4.6.1/bosTau7)) listed in Additional file 5: Table S2. A total of 20 ng DNA of the Siberian roe deer with B chromosomes (CPY_d), flow sorting-derived CPY B chromosome-specific library and cattle genomic DNA were used as templates. The PCR program included: 94°С for 2 minutes; 30 cycles of 94°С (15 s), Х°С (30 s), 72°С (30 s); and a final extension at 72°С for 5 minutes, where Х corresponds to the respective annealing temperature for each pair of primers. PCR products were analyzed on a 1.5% agarose gel.

### qPCR

The fragment of the *PTGFR* gene amplified using the NBF and NBR primers (Additional file 6: Table S3) on cattle genomic DNA was used as a reference fragment for the real time PCR calibration. The primer sequences were designed from the genomic regions conserved between cattle and the Siberian roe deer. Real time PCR was performed using standard curve methods [51] using a C1000 Thermal Cycler (BioRad). C1000 manager software was utilized for the analysis of the results.

The copy number of regions was estimated in the genomes of cattle and Siberian roe deer with different numbers of B chromosomes. Each primer pair was tested by non-template PCR to check the primer-dimer formation in the reactions. A final dissociation step was always performed at the end of each PCR to identify the unique and specific amplification of the target sequence. Additional file 6: Table S3 lists the primers used for real time PCR in the present study. The amplification reactions were run three times. Reactions contained 10 μl of DNA (0.2 μg), 5 μl of primers mix (final primer concentration of 1 mM each), and 10 μl of Power SYBR Green PCR Master Mix (Sintol, Moscow, Russia), in a final volume of 25 μl. Amplifications were carried out in a Thermal Cycler C1000 (BioRad). The reactions started with an initial step at 95°C for 5 minutes. The reaction proceeded with 40 cycles of 95°C for 15 s, 60°C for 20 s and 72°C for 30 s. A final dissociation step was always performed to control the amplicons melting curves.

### Sequencing

B-specific cDNA clones were sequenced with standard M13 primers using DTCS Quick Start Master Mix and CEQ 2000 sequencer (Beckman-Coulter) in the Department of Veterinary Medicine (Cambridge, UK). PCR products for sequence analysis of *FPGT*, *TNNI3K* and *LRRIQ3* genes were generated using primers based on cattle chromosome 3 region (UCSC genome browser on Cow, October 2011 (Baylor Btau_4.6.1/bosTau7)) (listed in Additional file 7: Table S4). For comparative analysis of non-B chromosomal and B chromosome-specific copies we amplified the segments from B chromosome-specific libraries, DNA of individuals lacking Bs and microdissected autosomes. Sequencing was carried out in the Interinstitutional center of DNA sequencing at the Siberian Branch of Russian Academy of Sciences (Novosibirsk, Russia) using an ABI3130×1 Genetic Analyzer (Applied Biosystems Inc., CA, USA) with ABI Big Dye kit according to a standard protocol. Sequence analysis was accomplished using Sequence scanner V1.0 software (Applied Biosystems), Blast and Blat alignment tools [52,53].

### Microdissection

We microdissected several copies of the largest autosome (CPY1) and B chromosomes as described earlier [54] with some modifications: after incubation in the collection drop with proteinase K and SDS, the sample was transferred to water, denatured and adjusted with all reagents necessary for PCR with T2 primers (Additional file 5: Table S2). In all, 40 cycles were performed (controlled with samples containing other autosomes and total genomic DNA) revealing fragments of interest. The fragments were directly sequenced after ExoSap treatment.

### Accession numbers

The GenBank [55] accession numbers for DNA sequences are JN871269 to JN871295.

## Abbreviations

BAC: Bacterial artificial chromosome; CBCL: B chromosome-specific cDNA library; FISH: Fluorescence *in situ* hybridization; SHAC: Selection of hybrids by affinity capture.

## Competing interests

The authors declare they have no competing interests.

## Authors’ contributions

VAT conceived the project and designed the experiments. VAT, PVD and PCMO’B performed the experiments. VAT, PVD, DML, PCMO’B, PLP, FY, MAF and ASG wrote the manuscript. All authors read and approved the final manuscript.

## Supplementary Material

Additional file 1 **Nucleotide and amino acid sequence of Siberian roe deer (*Capreolus pygargus *(CPY)) CPY_a *FPGT *three exons, CCA (from *Capreolus capreolus*) and CPY_d *TNNI3K *first exon and nucleotide sequence of c6h cDNA.** Click here for file

Additional file 2: Table S1 The results of bovine bacterial artificial chromosome (BAC) clone mapping on chromosomes of the Siberian roe deer. Click here for file

Additional file 3: Figure S1 Fragments of Siberian roe deer (*Capreolus pygargus* (CPY)) CPY1 used for quantitative polymerase chain reaction (qPCR). BTA3 is the segment of cattle chromosome 3 from the Btau_4.6.1. Click here for file

Additional file 4: Figure S2 Flow-karyotype of the Siberian roe deer (*Capreolus pygargus*). ‘B’ indicates a peak containing B chromosomes. Click here for file

Additional file 5: Table S2 Primers used for polymerase chain reaction (PCR) mapping of the Siberian roe deer (*Capreolus pygargus* (CPY)) CPY1 region on B chromosomes. ‘В’: presence (+) or absence (−) of the PCR product using the flow-sorted Siberian roe deer B chromosome-specific library. ‘СРY_d’: presence of the PCR product using the Siberian roe deer genomic DNA (CPY_d) genomic DNA. ‘ВТА’: presence of the PCR product using the bovine genomic DNA. Click here for file

Additional file 6: Table S3 Primers used in quantitative real-time polymerase chain reaction (PCR). Click here for file

Additional file 7: Table S4 Primers used for sequencing of *FPGT*, *TNNI3K* and *LRRIQ3* gene fragments. Click here for file
