# Supplementary material for: Transcription of a protein-coding gene on B chromosomes of the Siberian roe deer (Capreolus pygargus)
Source: BMC Biol. 2013 Aug 6;11:90. doi: 10.1186/1741-7007-11-90 (PMC3751663; doi:10.1186/1741-7007-11-90)
Supplement: Additional file 1 — Nucleotide and amino acid sequence of Siberian roe deer (Capreolus pygargus (CPY)) CPY_a FPGT three exons, CCA (from Capreolus capreolus) and CPY_d TNNI3K first exon and nucleotide sequence of c6h cDNA. [file 1741-7007-11-90-S1.doc]

Nucleotide and amino acid sequence of CPY_a *FPGT* three exons (the nucleotide/amino acid residue mutated in B chromosomes A→G (K83→R83) is yellow marked):

Exon 1: ATGGACGCTGAAAGTAGACCTGCCGGCGAATCTTTGCGAGAAGCCACCCAGCGAAGGTTGCGGAGGTTTTCAGAGCTTAGAGGT

Exon 2:

GGCAAATCAGTGGCACCTGGAGAATTCTGGGACATTGTTGTAATAACAGCAGCTGATGAAAAACAGAAACTTGCTTATAAGCAACAGCTTTCAGAAAAGCTGAAAAAAAAGGAGTTACCCCTTGGAGTTCAATATCATGTTTTTGCTGATCCTTCTGGAGCCAAAATT

Exon 3:

GGAAATGGAGGAGCAACCCTTTGTGCCCTTCGATGTTTGGAAAAGCTTTATGGAGATGAATGGAATTCTTTTACCATCCTATTAATTCATTCT

MDAESRPAGESLREATQRRLRRFSELRGGKSVAPGEFWDIVVITAADEKQKLAYKQQLSEKLKKKELPLGVQYHVFADPSGAKIGNGGATLCALRCLEKLYGDEWNSFTILLIHS

Nucleotide sequence of CCA and CPY_d *TNNI3K* first exon:

GATGAATGGAAGAAGAAAGTCAGTGAATCTTATGTTATTGTAATAGAAAGATTAGAAGATGACCTGCAGATCAAAGAAAAAGAACTTACAGAACTGAGGCATATATTTGGG

Amino acid sequence encoded by *TNNI3K* first exon of CCA and CPY_d:

DEWKKKVSESYVIVIERLEDDLQIKEKELTELRHIFG

Nucleotide sequence of c6h cDNA:

GGGTCACCAGTAAATGTGCTCTGTCTTGTAATATGGTTACTAAACATAATGAAATATATTTTTGGGAGAAATCCATTTTCCCCTCTGAAAGACTCCACAATCTGTTCCCAAATGCCATTGTCATAATCACCCCCACCATCACCTCCCTTTA
