# Supplementary material for: Transcription of a protein-coding gene on B chromosomes of the Siberian roe deer (Capreolus pygargus)
Source: BMC Biol. 2013 Aug 6;11:90. doi: 10.1186/1741-7007-11-90 (PMC3751663; doi:10.1186/1741-7007-11-90)
Supplement: Additional file 2: Table S1 — The results of bovine bacterial artificial chromosome (BAC) clone mapping on chromosomes of the Siberian roe deer. [file 1741-7007-11-90-S2.doc]

| Bovine ВАС clones | The coordinates on cattle chromosome 3 (Btau_4.6.1), bp | Genes present in the BAC clone | FISH signals on CPY B chromosomes | FISH signals on CPY1 | FISH signals on BTA3 |
| --- | --- | --- | --- | --- | --- |
| CH240-131I21 | 74 245 590-74 473 590 | *TNNI3K (*partial) | - | + | + |
| CH240-10H15 | 74 503 590-74 630 590 | *TNNI3K* | + | + | + |
| CH240-444I8 | 74 611 590-74 806 590 | *FPGT*  *FGD5-AS1* *LRRIQ3* (partial)  *TNNI3K* | + | + | + |
| CH240-493Р4 | 74 795 590-74 990 590 | *LRRIQ3*  *RNPS1* | + | + | + |
| CH240-515С3 | 74 944 590-75 180 590 |  | + | + | + |
| CH240-454D22 | 76 240 590-76 385 590 |  | + | + | + |
| CH240-351I13 | 76 410 590-76 580 590 | *ARPC2* | + | + | + |
| CH240-385G2 | 76 693 590-76 980 590 |  | - | + | + |
