# Supplementary figures and images for: Transcription of a protein-coding gene on B chromosomes of the Siberian roe deer (Capreolus pygargus)
Source: BMC Biol. 2013 Aug 6;11:90. doi: 10.1186/1741-7007-11-90 (PMC3751663; doi:10.1186/1741-7007-11-90)

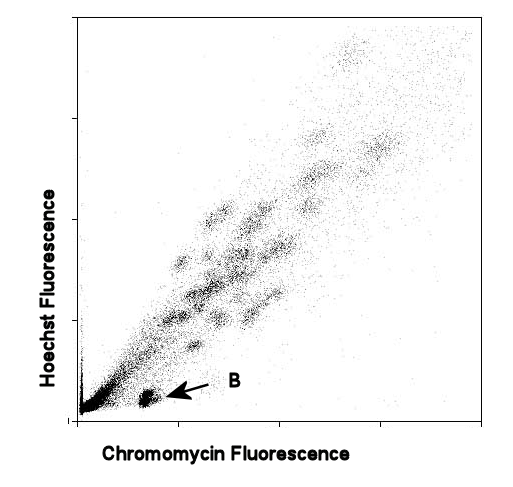

Supplement: Additional file 4: Figure S2 — Flow-karyotype of the Siberian roe deer (Capreolus pygargus). ‘B’ indicates a peak containing B chromosomes. [file 1741-7007-11-90-S4.tiff]
