# Supplementary material for: Transcription of a protein-coding gene on B chromosomes of the Siberian roe deer (Capreolus pygargus)
Source: BMC Biol. 2013 Aug 6;11:90. doi: 10.1186/1741-7007-11-90 (PMC3751663; doi:10.1186/1741-7007-11-90)
Supplement: Additional file 5: Table S2 — Primers used for polymerase chain reaction (PCR) mapping of the Siberian roe deer (Capreolus pygargus (CPY)) CPY1 region on B chromosomes. ‘В’: presence (+) or absence (−) of the PCR product using the flow-sorted Siberian roe deer B chromosome-specific library. ‘СРY_d’: presence of the PCR product using the Siberian roe deer genomic DNA (CPY_d) genomic DNA. ‘ВТА’: presence of the PCR product using the bovine genomic DNA. [file 1741-7007-11-90-S5.doc]

| № | Primer name | Primer sequence | Coordinates on the BTA 3 (Btau_4.6.1) | Size of the fragment, bp | В | СРУ_d | ВТА |
| --- | --- | --- | --- | --- | --- | --- | --- |
| 1 | T5F T5R | CTCGCTAAGAAGAACACTGTCC TGACCGCTGGACCACCAG | 74505791-74506060 | 270 | - | + | + |
| 2 | T159F T159R | GCATGTAGGGAAGAACCATG CAATCCCACTGGCAATGG | 74530162-74530471 | 310 | - | + | + |
| 3 | 5168F 5168R | TCACATTGCTAACTTGAAAGAGC CTGGCCATCCATGTTTCGC | 74538669-74538976 | 308 | - | + | + |
| 4 | 5182F 5182R | CTTAGCATGCAATGTATGCTCT ATCACAGCCTGAGACTGGC | 74553324-74553541 | 218 | + | + | + |
| 5 | T19LF T19LR | GGTGCAACATTCTATTGCCT ATCATTGCAGTAGATGTTGCC | 74600303-74600544 | 242 | + | + | + |
| 6 | 24F 24R | ATGCCTTCTTATGTATCTCGGTGG CAGGACAATGAAGATCACGTTCC | 74620585-74621427 | 843 | + | + | + |
| 7 | T4F T4R | GTGTCTACATTTTGCAAAGCAC TGTTGAAGGTTACATGAGTTGC | 74729283-74729552 | 270 | + | + | + |
| 8 | 35F 35R | CTTTACCCAAATATATGCCTCAGTTCT CTGATTAATTCCTTTCATGATTTCACC | 74729379-74730339 | 961 | + | + | + |
| 9 | T2F T2R | GGTAAAACCTGTAACAGCTAGAG AGGTGGAAACATACATAGTCCC | 74780426-74780761 | 336 | + | + | + |
| 10 | LR1F LR1R | CATGGAACAATCATAGAAGAGCT ACTGTGTATCGATAACCTAAGAGC | 74801521-74801841 | 321 | + | + | + |
| 11 | LR2F LR2R | TTCTCTATCTACATGACAATGGG GAATCTTTCAGGAAGATGCCAG | 74805587-74805807 | 221 | + | + | + |
| 12 | LR4F LR4R | GCAATTCCCTCTTCCTCCC CTTACATGTTTCCAGCGTGC | 74855858-74856045 | 188 | + | + | + |
| 13 | LR5F LR5R | GGAGCTCACTGTCCTCAAG CTAGTGTCCAATCAGAGACAGG | 74893978-74894236 | 259 | + | + | + |
| 14 | 5618F 5618R | AAGAGAATGAAAGCTGGTCTCT GCAGCAGCTTACAAGAAGAGA | 74989034-74989223 | 190 | + | + | + |
| 15 | 5709F 5709R | GTGATCTATCGCCTCACGGAAGG GGAGTAGAAGGGAGTGCCTGTGC | 75080444-75080987 | 544 | + | + | + |
| 16 | 5711F 5711R | GTGCCTAGATATCACAAACCACAGG GGCATCCTGTACCTCTTCACTTGAT | 75082566-75082885 | 320 | + | + | + |
| 17 | 5715F 5715R | CAGATACTGGCAGTATGACCAAG GTAGGTTTCTGAAGTTGCAAGGTAG | 75085756-75086158 | 403 | - | + | + |
| 18 | 5716F 5716R | GTCCAACATTGATCTAATCTGTACC AAAGAGCTACTGTTTGCTAGATGC | 75087511-75087842 | 332 | - | + | + |
| 19 | 5768F 5768R | GTGGGATTGGAGTCAGGAGG CAAAGCTCACTCCTGTTAGCAGAT | 75139555-75140172 | 618 | + | + | + |
| 20 | 5863F 5863R | CATGAATGGATCCCAAGGC TGACTGTGACCCTTCACTCCC | 75233849-75234411 | 563 | - | + | + |
| 21 | 759F 759R | CAAGAGGGACACTGAGGTCAGC CCCACACGGTGGCACATG | 75303594-75304112 | 519 | + | + | + |
| 22 | 604F 604R | TAGATGTGTTCTGCAAGATGGGGA TGTCATCATCAAAATGCCAGGATC | 75412211-75412381 | 171 | + | + | + |
| 23 | 6103F 6103R | GGTGGCCTTCTCCTCAGGTC GAAGCTCGGGAACCTCTGGAG | 75473859-75474135 | 277 | + | + | + |
| 24 | 6135F 6135R | GCTTCTGCTTCCTCTTTGTCCAC TGTTGCCTGGTAACCAACTTGC | 75505897-75506106 | 210 | + | + | + |
| 25 | 6179F 6179R | CAGTAGCCACTCTAGAAGATTCTGG CTGTTCCTGCCTTCCAGAGAA | 75550251-75550616 | 366 | + | + | + |
| 26 | 762F 762R | CAGCACATGCATCACTCAGGG GTCAGCCACCATGCAGAGCC | 75571319-75571575 | 257 | - | + | + |
| 27 | 7620F 7620R | TCGTCAAGCTAGGTTAATGGTGG CCTCATGGCTCTCTTGATCAGTT | 75571678-75572134 | 457 | + | + | + |
| 28 | 6232F 6232R | CTGGTTCTACCTACCCTACTCAACA ATTTAGCCAAGTCTGTTCTGGGA | 75603320-75603498 | 179 | + | + | + |
| 29 | 6307F 6307R | CAGGCAATGAAGGGTGGAG CGTCATTCAGCATGAGGCAA | 75677761-75678214 | 454 | + | + | + |
| 30 | 766F 766R | GAGATCTTCCTGGCAGTTGCTC CATGTCATCAGCCTGGGTGAG | 75686759-75687155 | 397 | - | + | + |
| 31 | 6343F 6343R | GCTTCTCTCTTTGTTGCCTGC GGCTGGTTTTAGCTAATCAAGCT | 75714280-75714654 | 375 | - | + | + |
| 32 | 6453F 6453R | TTCACAGGGTTGGAAGCAGTC TGCTTGCACCACTGCTGTC | 75823761-75824269 | 509 | + | + | + |
| 33 | 764F 764R | CACAGATCCTTCCCCTCTCCC GTGGTGCCCAATAGCACAGC | 75830409-75830896 | 488 | + | + | + |
| 34 | 6685F 6685R | CCCACTAACTACCATAGCAGATACC GTCTCAAGCCATCTGGAAGAGTC | 76001587-76001952 | 366 | + | + | + |
| 35 | 669F 669R | AGCTTTCGTAGTTCAGGGGCAGT AATGCCCTGAATAATATTGCCAGG | 76008917-76009510 | 594 | + | + | + |
| 36 | 6719F 6719R | CCCTAAGGAAACAGAGGGGG GTCATGGAGACTTCATCTTGGG | 76035003-76035276 | 274 | + | + | + |
| 37 | 6752F 6752R | TACACTTCTCAGTAAGAGCACAG GGAAACATGAGCTGATACAGTTC | 76068308-76068627 | 320 | + | + | + |
| 38 | 7152F 7152R | GGAGGGTCAAGTGGGGC ACCAACCAGGAATGAACCATG | 76468193-76468619 | 427 | + | + | + |
| 39 | 7186F 7186R | AAGGCCTCTGGACTTCTGTCC GCACTGAACAGGCATAATCCCTC | 76502073-76502262 | 190 | - | + | + |
| 40 | 725F 725R | CCAAGGCAACATGACAGGG GCCAACACTACCTGGTTGC | 76568504-76568884 | 381 | - | + | + |
