# Supplementary material for: Transcription of a protein-coding gene on B chromosomes of the Siberian roe deer (Capreolus pygargus)
Source: BMC Biol. 2013 Aug 6;11:90. doi: 10.1186/1741-7007-11-90 (PMC3751663; doi:10.1186/1741-7007-11-90)
Supplement: Additional file 6: Table S3 — Primers used in quantitative real-time polymerase chain reaction (PCR). [file 1741-7007-11-90-S6.doc]

| Primer name | Primer sequence | Coordinates on cattle chromosome 3 (Btau_4.6.1) | Fragment size, bp | Gene |
| --- | --- | --- | --- | --- |
| PT4RF  PT4RR | TCCATTTGGCAGTCAGCTT AATTTCCCATTTGTTAGAAGTTTC | 74730128-74730266 | 139 | *TNNI3K* |
| FPGTBF FPGTBR | CTTGCACTCCACATGCCAT AATTGCATCATTAGTGGTGCTTAT | 74775958-74776084 | 127 | *FPGT* |
| LR4F  LR4R | GCAATTCCCTCTTCCTCCC CTTACATGTTTCCAGCGTGC | 74855858-74856045 | 188 | *LRRIQ3* |
| NBF  NBR | TGTAGCTTTGCTGCCCATC AACCTATCTTCCCAGTCTTTGATT | 70104006-70104110 | 105 | *PTGFR* |
