# Supplementary material for: Transcription of a protein-coding gene on B chromosomes of the Siberian roe deer (Capreolus pygargus)
Source: BMC Biol. 2013 Aug 6;11:90. doi: 10.1186/1741-7007-11-90 (PMC3751663; doi:10.1186/1741-7007-11-90)
Supplement: Additional file 7: Table S4 — Primers used for sequencing of FPGT, TNNI3K and LRRIQ3 gene fragments. [file 1741-7007-11-90-S7.doc]

| Primer name | Primer sequence | Coordinates on cattle chromosome 3 (Btau_4.6.1) | Fragment size, bp |
| --- | --- | --- | --- |
| 40F  40R | TGAGATTCCAAATGTCTAAGCATGAC ACCTCGAATACAGAAGTTGCCATC | 74775862-74776728 | 867 |
| FPGT1F  FPGT1R | ATGTCTCTAAATCAGACTGCTTCCTA CCATCGATACCAGAATGCTCTACT | 74775586-74776213 | 628 |
| FPGT2F  FPGT2R | GGTCTACACACAGCATCAAACTCATAC ATGTGTCATAGCCTGTAAAGCATATTTAG | 74776647-74777283 | 637 |
| FPGT3F  FPGT3R | ACTCCTGCATTTGTGATCTTGCT GGGTCTGTTTCCTGTCATGCTT | 74775394-74775900 | 507 |
| T3F  T3R | GCCTGTACCTTTGCTCTCTGC AATGTCCCTGAGATATCAAGGG | 74779024-74779288 | 265 |
| T2F  T2R | GGTAAAACCTGTAACAGCTAGAG AGGTGGAAACATACATAGTCCC | 74780426-74780761 | 336 |
| T1F  T1R | GCCAATGTCCTAGGTCCGCA AGAGCTTCGGGGGCGTGG | 74782110-74782465 | 356 |
| PT4F  PT4R | CAGTACAAGTTTGCGTTGGTCTG TTCCTTTCATGATTTCACCCTTG | 74730098-74730331 | 234 |
| T4F  T4R | GTGATGCAAACAGGCAAGGC TCACTTGTATGTTATGAGCTGTGTCTAC | 74729166-74729572 | 407 |
| LR2F  LR2R | TTCTCTATCTACATGACAATGGG GAATCTTTCAGGAAGATGCCAG | 74805587-74805807 | 221 |
| LR4F  LR4R | GCAATTCCCTCTTCCTCCC CTTACATGTTTCCAGCGTGC | 74855858-74856045 | 188 |
